# Effect of Physical Activity and Nutrition Education on Body Mass Index, Blood Pressure and Biochemical Variables in Overweight and Obese Adolescents

**DOI:** 10.5334/aogh.3147

**Published:** 2021-01-25

**Authors:** Jeanne Grace, Chara Biggs, Alden Naicker, Sarah Moss

**Affiliations:** 1 University of KwaZulu-Natal, ZA; 2 North-West University, ZA

## Abstract

**Background:**

The majority of obese children lives in developing countries. The ten-fold increase in obesity during the last four decades necessitates the implementation of interventions to mitigate the longterm effect of obesity into adulthood. The study aimed to determine the impact of physical activity and nutrition intervention on the body mass index (BMI), blood pressure and selected biochemical factors of overweight and obese children aged 13.0 to 16.1 years from eThekweni, South Africa.

**Methods:**

Participants (N = 41) with a BMI >85^th^ percentile were included in the 10-week controlled trial of physical activity and nutrition education intervention. Baseline and end measurements included BMI, blood pressure, and fasting biochemical variables (glucose, cholesterol, insulin resistance and alanine aminotransferase). BMI was classified according to the WHO BMI z-scores. The 10-week intervention entailed combined aerobic and resistance exercises supervised twice a week together with a once a week nutrition intervention. Participants performed additional unsupervised aerobic exercises three times a week.

**Findings:**

Elevated systolic blood pressure (52%), low-density lipoprotein levels (29%), insulin (17%) and insulin resistance values (15%) were identified. The 10-week intervention programme significantly decreased BMI (30.8 ± 5.4 kg/m^2^ to 29.8 ± 5.7 kg/m^2^; p < 0.01), systolic blood pressure (125.9 ± 15.7 mmHg to 115.2 ± 12.4 mmHg; p < 0.05), and low-density lipoprotein cholesterol (2.63 mmol/L to 2.37 mmol/L; p < 0.05). Controlling for pre-testing variables as covariates, additional ANCOVA analysis highlighted significantly lower BMI (M = 28.33, F = 7.88, p < 0.05) and BMI z-scores (M = 2.08, F = 4.99, p < 0.05) in the intervention group post-testing.

**Conclusion:**

A 10-week physical activity and nutrition education intervention in overweight and obese adolescents significantly reduced BMI and showed trends of a decrease in blood pressure and low-density lipoprotein cholesterol.

## INTRODUCTION

The prevalence of obesity among adults and children in both developed and developing countries has reached epidemic proportions [1,2]. Globally in 2013, the prevalence of overweight and obese children and adolescents in low- to middle-income countries was estimated at 12.9% for boys and 13.4% for girls with South African children and adolescents having the highest prevalence of overweight (22%) and obesity (11%) globally [1,3]. According to the World Health Organization (WHO), the majority of overweight and obese children live in developing countries with obese children usually becoming obese adults [4,5]. Adolescents from developed and developing countries are now affected by the obesity pandemic in a similar pattern [4]. An increase in overweight and obesity is linked to fatty liver disease, sleep apnoea, type 2 diabetes, asthma, hypertension, cardiovascular disease, high cholesterol levels, glucose intolerance and insulin resistance, menstrual abnormalities, impaired balance, and orthopaedic problems [6]. Hence, it is essential to determine the presence of these risk factors early in life for overweight and obese adolescents to implement preventative strategies [5]. In South Africa, almost 50% of the population lives with hypertension, which is considered to have its origin in childhood [7]. Early data indicate the prevalence of hypertension in South African children as between 7% and 22% [8].

The association of many comorbidities with overweight and obesity highlights the cost of obesity-related medical expenses estimated at $190.2 billion, with $14 billion in direct medical costs for children [9]. Moreover, healthcare costs of childhood obesity broaden when considering the economic burden of a lifetime of weight-related healthcare needs underscoring the importance of implementing obesity prevention and treatment programs in children to reduce obesity-related externalities [10].

Although most of the physical health conditions such as hypertension and diabetes associated with childhood obesity are preventable and can disappear when a child or adolescent reaches a healthy weight, some continue to have negative consequences throughout adulthood [2,6]. The most successful interventions to treat overweight and obese children younger than 18 years of age are multidimensional, combining diet and physical activity in school, community, and home life settings [4,11].

The application of formative research before the implementation of interventions, as well as integrating interventions into already existing lifestyle school programs and structures, ensure maximum reach and sustainability [12]. Studies within the South African context investigating the effect of physical activity and diet on overweight and obese children within the school setting assessed self-perception as the outcome measure [13].

Researchers who implemented dietary, and or physical activity interventions in overweight and obese adolescents, reported improvements in body composition [11,14,15], decrease in systolic blood pressure [16], alanine transaminase (ALT) [17], glucose, insulin, homeostatic model assessment of insulin resistance (HOMA-IR) [18,19], glycated haemoglobin (HbA1c), total cholesterol, low-density lipoprotein-cholesterol (LDL-C), and triglyceride levels, and an increase in high-density lipoprotein cholesterol (HDL-C) levels [15,20]. Despite the benefits of dietary and or physical activity interventions on anthropometrical, selected physiological and biochemical parameters in overweight and obese adolescents in high-income countries, there is minimal evidence for interventions aimed at obese and overweight adolescent children from low-to-middle income countries (LMIC) like South Africa. To address the critical gap that will enable effective management of overweight and obese adolescents globally; the study aimed to determine the effect of a combined physical activity and nutrition education intervention program on body mass index (BMI), blood pressure and selected biochemical variables in overweight and obese South African adolescents from the KwaZulu-Natal area.

## METHODS

### STUDY DESIGN AND SETTING

A quasi-experimental pretest-posttest 10-week intervention study was conducted in two semi-private schools within the eThekwini Municipality, South Africa. The municipality encompasses several neighbouring settlements with a population of 3,442,361 in a density of 2,291.31 km^2^, according to the 2007 community survey. Black Africans (74%), White Africans (27%), Indian (7%) and Asians (17%) primarily inhabit the area [21].

### SAMPLING

Sixteen of the 30 randomly identified semi-private schools in the eThekwini Municipality were invited to participate in the study. Nine-hundred learners aged 13–16 years were recruited from grades 8 and 9 from two schools. Informed consent was obtained from 656 (72.3%) parents/guardians of the learners (Phase 1). Eligible participants were identified by measuring their height and weight to calculate BMI. Of these 247 (32.9%) learners, of which 153 (61.9%) were boys, and 94 (38.1%) were girls, had a BMI ≥85^th^ percentile and were approached for inclusion in the intervention study (Phase II). Assent was given by 129 (52.2%) learners, of which 48 (37.2%) were boys, and 81 (62.7%) were girls. These participants were then conveniently allocated to either the intervention (104/129, 80.6%) or control group (25/129, 19.4%). The allocation was based on timetable clashes and availability of transport. In total 41 (31.8%) learners participated in the 10-week intervention (initially, the duration of the intervention was 16 weeks, but due to the non-adherence of the participants, the intervention was stopped after ten weeks instead of 16 weeks), which consisted of the physical activity and nutritional education group (PAN) (7/41, 17.2% boys, 15/41, 36.4% girls) and the control group (CG) (11/41, 26.8% boys, 8/41, 19.6% girls).

The study was approved by the Universities’ Biomedical research and ethics committee (BFC499/17). All procedures performed in this study involving human participants were in accordance with the 1964 Helsinki Declaration and its later amendments or comparable ethical standards.

### MEASUREMENTS

For Phase I, all grade 8 and 9 learners’ height and weight were measured at school in groups of between 100 and 150 learners per day over three days. Phase II of the study commenced one week after the identification of eligible learners. On arrival at school in the morning after a 9-hour fast, blood pressure (BP) was measured after which a nurse collected the 20 ml blood sample from the antecubital vein. The learners then received light refreshments, after the demographic information was completed. Phase II data were collected at baseline and ten weeks post-intervention.

### ANTHROPOMETRICAL MEASURES

Height (scale SECA model 217) and weight (scale AND UC-321, A&D Medical) was measured according to the standards of the International Society for the Advancement of Kinanthropometry (ISAK) [22]. All measurements were repeated twice, with a third measurement if the first two measurements in weight differed by more than 100 g or the height by 0.1 cm. The nearest two measurements were averaged and used in the data analyses. Weight was measured in school uniform without blazers and shoes. A weight of 400 g for girls and 800 g for boys was subtracted from the measurement to compensate for the school uniform. The measurements were entered into the WHO AnthroPlus software to calculate the BMI as weight/height^2^ (kg/m^2^) and to plot the BMI on the growth charts [23]. The WHO BMI-based definitions of overweight (≥85^th^ percentile), and obesity (≥97^th^ percentile) was used to identify learners eligible for inclusion in Phase II [24].

The BMI z-scores were used as an outcome measurement of the intervention as changes could be more accurately monitored from pre to post-intervention. Overweight was defined as >+1SD and obesity as >+2SD [24].

### BIOCHEMICAL MEASURES

Biochemical analyses were performed on the plasma prepared from the collected fasting blood samples. Samples were collected in VACUCARE blood collection tubes (EREZ labmed, Midrand, South Africa). The tubes were stored at between 2°C to 8°C and the analysis completed within 8 hours of collection. Fasting serum insulin concentrations were assessed using a chemiluminescent microparticle immunoassay (Abbott Architect System, Irving, TX, USA). Plasma glucose and glycated haemoglobin (HbA1c) were measured using Vitros DT60 II Chemistry Analyser (Ortho-Clinical Diagnostics, Rochester, NY, USA) with VITROS reagents and control. Elevated HbA1c was defined ≥6.2% [25]. Plasma triglycerides (TG), high-density lipoprotein cholesterol (HDL-C), and low-density lipoprotein cholesterol (LDL-C) concentrations and alanine aminotransferase (ALT) were determined with an immunoradiometric assay (Active Human Leptin IRMA, DSL-23100, Diagnostic System Laboratories Inc., Webster, TX, USA).

The 2018 AHA/ACC/AACVPR/AAPA/ABC/ACPM/ADA/AGS/APA/ASPC/NLA/PCNA Guideline on the Management of Blood Cholesterol for children was used to define abnormal cholesterol levels (HDL-C: ≤1.2 mmol/L, LDL-C: ≥2.8 mmol/L and TG as ≥1.0 mmol/L) [26]. LabCorp’s ALT reference intervals as >25 U/L for females aged 12–17 years and >31 U/L for males aged 12–17 years were applied [27]. Fasting insulin levels ≥20 mU/ml were considered hyperinsulinemic levels [28]. Insulin resistance was computed using the homeostatic model assessment of insulin resistance (HOMA-IR) and assessed using the following formula: HOMA-IR = (fasting insulin [μIU/mL] × fasting glucose [mmol/L])/22.5 [29]. Insulin resistance was defined as HOMA-IR ≥3.4 [30].

### BLOOD PRESSURE

Blood pressure was measured to the nearest two mmHg using an automated Omron sphygmomanometer (Omron Healthcare Europe BV) and an appropriate size cuff for each participant as per the recommendations of the American Academy of Paediatrics [31]. The average of two readings, taken two minutes apart, was used in data analyses. The Clinical practice guidelines for screening and management of high blood pressure in children and adolescents of the American Academy of Paediatrics (AAP) was used to classify systolic and diastolic blood pressure: <90^th^ percentile as a normal systolic/diastolic blood pressure, >90^th^ and <95^th^ as elevated systolic/diastolic blood pressure and ≥95^th^ as Stage 1 systolic/diastolic hypertension, and ≥95^th^ percentile + 12 mmHg as Stage 2 systolic/diastolic hypertension [31]. The American Academy of Paediatrics calculator, which is based on the AAP’s 2017 Clinical Practice Guidelines, was used to classify the blood pressure into normal, elevated, Stage 1 and Stage 2 hypertension [31].

### INTERVENTION PROGRAMS

#### Supervised physical activity program

Following the American College of Sports Medicine guidelines [32], participants attended group classes, presented by a biokineticist (clinical exercise physiologist) twice a week during an extracurricular time slot. The interventions consisted of 20 (two days per week for ten weeks) exercise sessions. During the intervention, adherence to the interventions was monitored by recording the rate of perceived exertion (RPE) during each session. The Borg RPE scale is a self-report index that reflects the perceived exercise intensity. The intervention aimed at attaining an exercise intensity of between 60%–80% of the participant’s heart rate reserve (HRR). The corresponding RPE scale is a score of between 12–14 out of 20 [32]. Participants that attended less than 13 of the sessions (65%) during the ten weeks were excluded from the data analyses.

Each session began with five minutes of warm-up (stretching and flexibility), followed by 10 minutes of resistance training, 20 minutes of aerobic training and five minutes of cool down. The exercise program was split into three phases, each consisting of five different exercises for each phase, to ensure metabolic adaptations. Participants followed an individualised progressive resistance training program: Phase I (week 1–4), three sets of 30-second durations of five exercises at an RPE of 14–15; Phase II (week 5–7) three sets of 30-second durations of five exercises (RPE 15–16); Phase III (week 8–10) three sets of 30-second durations of five exercises (RPE 16–17) using the participant’s body weight as the primary form of resistance to encourage home-based exercising. The overhead press was the only exercise that required a free weight, which was a school bag. The exercises targeted the following muscle groups: upper back, shoulders, chest, quadriceps, hamstrings, abdominals and calves. The aerobic exercises were developed in conjunction with the inputs of the participants and consisted of walking, jogging, dancing, step-up exercises, tag, modified shumpu (a traditional African game), and ball games.

#### Unsupervised physical activity program

The participants were expected to perform an additional three days of unsupervised physical activity to the two supervised intervention sessions. The aim was to increase their physical activity and decrease sedentary behaviours. The prescribed unsupervised sessions consisted of 30 sessions (three days a week for ten weeks). Participants reporting less than 20 sessions after ten weeks (67% adherence), were excluded from the data analysis. Adherence was monitored by self-reporting of activities and RPE to the exercise specialist at the supervised sessions. The participants were advised to perform the aerobic program, consisting of walking, jogging, dancing, tag, modified shumpu and step-up exercises, at an exercise intensity of 60%–70% of heart rate reserve (HRR). Additionally, to ensure the required heart rate (HR) was reached, the participant’s HR (corresponding with the intensity of exercise needed to be calculated beforehand) had to correspond with their RPE scale.

#### Nutrition education program

A registered dietitian individually discussed information surrounding food consumption habits with each participant and highlighted specific issues such as excessive fat and/or sugar consumption and/or under consumption of fruit and vegetables. The participants then attended a weekly culturally sensitive interactive nutrition curriculum for one hour over 10-weeks, designed to encourage weight loss. The emphasis of the nutrition intervention was on healthy eating and a non-diet approach. The portion size was decreased by teaching the participants to identify hunger and eat only when hungry, savour their food properly, focus only on food while eating, and time their meals to curb their eating rate. The South African Food Based Dietary guidelines were used as the basis to improve the nutritional content of their diet [33]. Topics included the importance of curtailing the intake of sugar and the hidden sugar content of food and drinks, reducing fat intake, and identifying high-fat foods. The daily inclusion of at least five fruit and vegetables were promoted. Emphasis was placed on the role of exercise in weight loss and control. The participants were weighed weekly under the supervision of a registered dietitian and an attendance register was taken to monitor adherence. The results of those who attended fewer than six sessions over the ten weeks (60% adherence) were excluded from the data analysis.

### STATISTICAL ANALYSIS

Data were analysed using the Statistical Package for the Social Sciences (SPSS, version 20.0, Chicago. IL, USA). The primary analysis was “intention-to-treat” and included all participants that were allocated to the control or intervention groups. Descriptive statistics were performed to describe the participants at baseline by reporting the frequencies for categorical data and means and standard deviations for continuous variables. The intervention’s effect was determined with an analysis of covariance (ANCOVA) for the differences between the control and PAN group for variables that did not violate ANCOVA assumptions. Pre-intervention values were used as covariates. Where assumptions were violated, an independent t-test was conducted on the difference scores. The statistical analysis was considered significant when the probability level was <0.05.

## RESULTS

**Figure 1:**
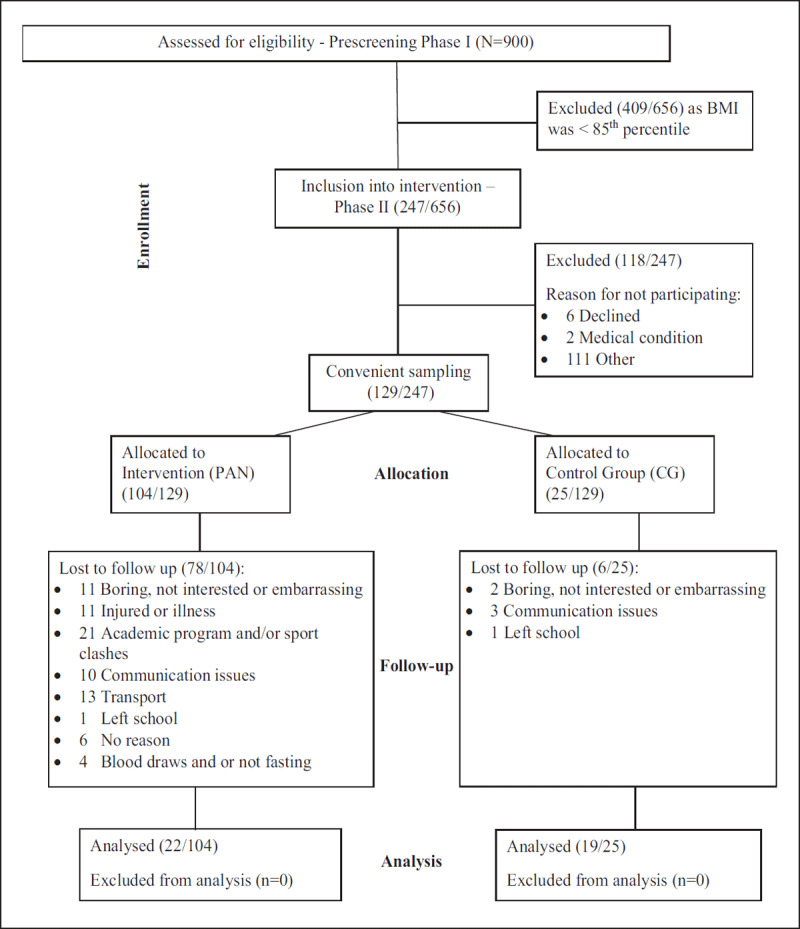
Consort diagram showing the participant’s flow through the study.

The Consort diagram shows the participant’s flow throughout the study with all reasons for learners’ exclusion and abandonment of the intervention (***Figure 1***). It is of particular interest that two girls were excluded as they were identified on screening with previously undiagnosed diabetes mellitus. Learners in the control group were asked to maintain their current activities of daily living (ADL).

The participants’ demographic information (***Table 1***) indicates no significant difference in age, height, and weight between the PAN and CG during pre-intervention testing. However, as displayed in ***Table 3***, the participants’ average BMI-for-age placed them at >+2 z-score for both girls and boys classifying them as obese when adjusting for age [24]. The majority in the PAN group (77.3%) and CG (68.4%) were of Black ethnicity with 68.2% and 42.1% respectively from the PAN group and CG being girls and 31.8% and 57.9% being boys (***Table 1***).

**Table 1:** Demographic characteristics of the participants in the physical activity and nutrition (PAN) group vs the control group (CG).


	N	PAN (M ± SD)	N	CG (M ± SD)

Age (years)	22	14.5 ± 0.7	19	13.9 ± 0.6

Weight (kg)	22	79.9 ± 14.9	19	73.6 ± 16.9

Height (cm)	22	161.2 ± 9.5	19	163.4 ± 10.4

Ethnicity (%)	22	100	19	100

Black	17	77.3	13	68.4

Indian	2	9.1	0	0

Caucasian	3	13.6	6	31.6

Gender (%)	22	100	19	100

Male	7	31.8	11	57.9

Female	15	68.2	8	42.1


Classification of the blood pressure according to the normal cut-off points, indicated that 52% (n = 21) of the participants presented with raised systolic blood pressure (SBP) levels pre- versus 34% (n = 14) post-testing while 22% (n = 9) had raised diastolic blood pressure (DBP) versus 15% (n = 6) post-testing. Of the participants, 29% (n = 12) had raised LDL-C levels compared to 17% (n = 7) post-testing with 24.3% (n = 10) whose HDL-C levels were too low and 12% (n = 5) had raised triglycerides levels. Elevated fasting insulin levels were present in 17% (n = 7) of the participants pre-testing compared to (7%) (n = 3) post-testing, and 15% (n = 6) were insulin resistant pre-testing compared to 12% (n = 5) post-testing (***Table 2***).

**Table 2:** Prevalence of abnormal blood pressure and biochemical measurements (N = 41).


VARIABLE	PRE (%)	POST (%)

Systolic blood pressure		

Normal (<90^th^ percentile)	20 (48)	27 (66)

Elevated (≥90^th^–<95^th^ percentile)	7 (17)	6 (15)

Hypertension stage 1 (≥95^th^ percentile)	8 (20)	7 (17)

Hypertension stage 2 (≥95^th^ percentile + 12 mmHg)	6 (15)	1 (2)

Diastolic blood pressure		

Normal (<90^th^ percentile)	32 (78)	35 (86)

Elevated (≥90^th^–<95^th^ percentile)	1 (2)	0 (0)

Hypertension stage 1 (≥95^th^ percentile)	8 (20)	5 (12)

Hypertension stage 2 (≥95^th^ percentile + 12 mmHg)	0 (0)	1 (2)

Blood pressure		

Normal (<90^th^ percentile)	17 (41)	24 (59)

Elevated (≥90^th^–<95^th^ percentile)	8 (20)	6 (15)

Hypertension stage 1 (≥95^th^ percentile)	10 (24)	10 (24)

Hypertension stage 2 (≥95^th^ percentile + 12 mmHg)	6 (15)	1 (2)

ALT (Males: >31, Female: >25 U/L)	2 (5)	2 (5)

HDL-C (≤1.2 mmol/L)	10 (24)	9 (23)

LDL-C (≥2.8 mmol/L)	12 (29)	7 (17)

TG (≥1.0 mmol/L)	5 (12)	3 (7)

Insulin (≥20 mU/ml)	7 (17)	5 (12)

HOMA-IR (≥3.4)	6 (15)	5 (12)


ALT – Alanine aminotransferase; HDL-C – High-density lipoprotein cholesterol; LDL-C – Low-density lipoprotein cholesterol; HOMA-IR – Homeostatic model assessment of insulin resistance; TG – Triglycerides.

As displayed in ***Table 2***, there were only two participants with raised ALTs which negated statistical analysis, although it is interesting to note the one in the PAN group was slightly lowered while the one in the CG doubled post-intervention. When only considering those with a raised SBP pre-testing, the post scores were significantly higher (p > 0.05) for the CG (M = 129.3) than for the PAN group (M = 120.2), although there was no significant difference in pre- and post-diastolic values. When considering only those with a low HDL-C, high TG and LDL-C or a raised HOMA, no effect was found in the PAN group.

As displayed in ***Table 3***, the BMI, blood pressure and biochemical values did not differ significantly across the two groups before the intervention program. Before the intervention program, the SBP of the PAN group (125.9 ± 15.7 mmHg) was elevated [31]. In contrast, all the mean biochemical values for both groups were within the clinically specified normal ranges (***Table 3***). Significant decreases pre- to post-testing were noted in the PAN group for BMI z-score (p < 0.0005), systolic blood pressure (SBP) (p < 0.05) and LDL-C (p < 0.05) with a significant increase in the HbA1c values of the PAN group (p < 0.05), and the CG (p < 0.0005) (***Table 3***). Although not significant, it is notable that the rest of the CGs biochemical values post-testing showed no change from baseline to post compared to the PAN group that showed improvement post-testing except for a significant increase in the HbA1c of the PAN group post-testing (***Table 3***).

**Table 3:** BMI, blood pressure and biochemical characteristics pre and post a 10-week physical activity and nutrition intervention compared to a control group.


VARIABLE	GROUP	N	PRE (M ± SD)	POST (M ± SD)

BMI (kg/m^2^)	PAN	22	30.8 ± 5.4	29.8 ± 5.7**

	Control	19	27.6 ± 5.3	27.7 ± 5.29

BMI z-score	PAN	22	2.46 ± 0.79	2.24 ± 0.89**

	Control	19	2.10 ± 0.82	2.03 ± 0.83

Systolic Blood Pressure (mmHg)	PAN	22	125.9 ± 15.7	115.2 ± 12.4*

	Control	19	117.3 ± 17.5	114.9 ± 13.9

Diastolic Blood Pressure (mmHg)	PAN	22	76.1 ± 8.1	73.3 ± 7.3

	Control	19	72.3 ± 7.8	70.0 ± 10.7

ALT (U/L)	PAN	22	18.1 ± 6.1	16.9 ± 5.6

	Control	17	18.9 ± 9.9	22.1 ± 22.4

Triglycerides (mmol/L)	PAN	19	0.74 ± 0.42	0.69 ± 0.29

	Control	15	0.64 ± 0.94	0.64 ± 0.11

HDL (mmol/L)	PAN	19	1.34 ± 0.29	1.32 ± 0.26

	Control	15	1.44 ± 0.42	1.44 ± 0.36

LDL (mmol/L)	PAN	19	2.63 ± 0.66	2.37 ± 0.49*

	Control	15	2.25 ± 0.69	2.36 ± 0.73

Glucose (mmol/L)	PAN	19	4.63 ± 0.42	4.63 ± 0.44

	Control	15	4.41 ± 0.43	4.45 ± 0.44

HbA1c (%)	PAN	22	5.4 ± 0.35	5.6 ± 0.44*

	Control	17	5.38 ± 0.27	5.57 ± 0.27^

Insulin (mU/ml)	PAN	21	13.95 ± 5.48	12.82 ± 4.10

	Control	15	10.39 ± 5.55	13.30 ± 5.21

HOMA-IR	PAN	19	3.01 ± 1.22	2.72 ± 0.95

	Control	15	2.06 ± 1.16	2.79 ± 1.01


M – Mean; SD – Standard deviation; ALT – Alanine aminotransferase; HDL-C – High-density lipoprotein cholesterol; LDL-C – Low-density lipoprotein cholesterol; HOMA-IR – Homeostatic model assessment of insulin resistance. * p < 0.05, pre PAN vs post PAN; ** p < 0.01, pre PAN vs post PAN. ^ p < 0.01, pre CG vs post CG.

Controlling for pre-testing variables as covariates, additional ANCOVA analysis highlighted significantly lower BMI (M = 28.33, F = 7.88, p < 0.05) and BMI z-scores (M = 2.08, F = 4.99, p < 0.05) in the PAN group during post-testing between the PAN group and CG. Additional ANCOVA analysis, controlling for the pre-testing values as covariates, showed no significant differences post-testing between the PAN group and CG for blood pressure and the biochemical variables (p > 0.05) (***Table 4***).

**Table 4:** BMI, blood pressure and biochemical characteristics post 10-week values for the physical activity and nutrition- and control group after correcting for the pre values after correcting for the pre values.


VARIABLE	GROUP	N	ESTIMATED POST MEAN (SEM)	MEAN DIFF	F	95% CI LOWER	UPPER

BMI (kg/m^2^)	PAN	22	28.33 (0.25)	1.09*	7.88	0.30	1.87

	Control	19	29.42 (0.28)				

BMI z-score	PAN	22	2.08 (0.05)	0.16*	4.99	0.02	0.30

	Control	19	2.23 (0.05)				

Systolic Blood Pressure (mmHg)	PAN	22	113.2 (2.14)	4.31	1.65	–2.49	11.13

	Control	19	117.6 (2.52)				

Diastolic Blood Pressure (mmHg)	PAN	22	72.3 (1.62)	–0.94	0.14	–6.08	4.20

	Control	19	71.4 (1.90)				

ALT (U/L)	PAN	22	17.51 (1.96)	3.79	1.62	–2.25	9.82

	Control	17	21.34 (2.23)				

Triglycerides (mmol/L)	PAN	19	0.66 (0.07)	0.12	0.00	–0.11	0.34

	Control	15	0.78 (0.09)				

HDL (mmol/L)	PAN	19	1.36 (0.04)	0.03	0.56	–0.09	0.16

	Control	15	1.39 (0.05)				

LDL (mmol/L)	PAN	19	2.28 (0.09)	0.25	2.79	–0.03	0.53

	Control	15	2.53 (0.11)				

Glucose (mmol/L)	PAN	19	4.58 (0.09)	–0.07	0.23	–0.37	0.23

	Control	15	4.51 (0.11)				

HbA1c (%)	PAN	22	5.62 (0.06)	–0.04	0.17	–0.22	0.15

	Control	17	5.58 (0.07)				

Insulin (mU/ml)	PAN	21	12.77 (1.01)	0.56	0.12	–2.72	3.83

	Control	15	13.33 (1.21)				

HOMA-IR	PAN	19	2.63 (0.22)	0.31	0.70	–0.45	1.08

	Control	15	(0.29)				


SEM – Standard error of the mean; CI – confidence interval. ALT – Alanine aminotransferase; HDL-C – High-density lipoprotein cholesterol; LDL-C – Low-density lipoprotein cholesterol; HOMA-IR – Homeostatic model assessment of insulin resistance. * p < 0.05 post PAN vs post CG (ANCOVA).

## DISCUSSION

The present study demonstrated that in overweight/obese South African adolescent boys and girls, a combined physical activity and nutrition education program over ten weeks significantly improved their BMI and BMI z-scores. Although the systolic blood pressure and diastolic blood pressure decreased on average after the intervention, the decrease did not reach significance when adjusted for the control group. The improvements are reflected by a decrease in the number of participants presenting with hypertension and LDL-C concentrations. The prevalence of 59% participants presenting with raised blood pressure levels reflects the presence of risk factors in overweight or obese children. This prevalence is significantly higher than the 33.5% reported by a previous study in adolescents aged 13–17 years in high schools within a 10 km radius of Mthatha central business district, Eastern Cape in South Africa [34]. More recently, a study of Grade XII learners (Mean age = 17.8 years) from 15 schools in 5 different districts in KwaZulu-Natal reported 43.4% of the adolescents were hypertensive [35].

Since the participants were all either overweight or obese, improvements in BMI was expected, because various researchers who implemented nutrition and or physical activity interventions in overweight and obese adolescents, reported improvements in body composition [11,14,15]. Our results support researchers’ findings [16], who showed a significant decrease in systolic blood pressure and LDL-C after an exercise intervention. Conflicting results have been published with regards to other biochemical measurements in children after a physical activity intervention.

Other researchers report decreases in ALT [17], glucose, insulin, HOMA-IR [18,19], LDL-C, HbA1c and triglyceride concentrations with an increase in HDL-C concentrations after implementing nutritional and physical activity interventions [11,15,20]. Results from our population indicate contradictory findings to previous research. The reason for the difference in outcomes may be due to the previous studies reporting on interventions lasting up to six months, compared to the 10-week intervention of the current study [18,19]. Similarly, the duration of the lifestyle intervention program inclusive of physical activity, nutritional education and stress management implemented by Top et al. showed significant changes in obese adolescents’ LDL-C, HbA1c, triglycerides and HDL-C after six months [15]. Although Wang et al. established a significant change in the ALT levels of obese adolescents with physical activity and nutritional education program after only four weeks when obese participants with non-alcoholic fatty liver disease (NAFLD) were included in the study [17]. Differences reported by various researchers can be ascribed to differences in the study population’s characteristics.

Nonetheless, although the duration of the current intervention program was of shorter length compared to most previous studies, and only showed significant changes in BMI z-scores, systolic pressure and LDL-C, it is creating a sensitivity for researchers of systematic reviews and meta-analyses on childhood obesity prevention programs to consider interventions shorter than six months. Equally, policy-makers and program developers can also consider planning and implementing shorter interventions. However, researchers argued that six months might be too short a time to observe intervention effects on weight-related outcomes [11,16,36].

Together with statistical significance, it is also important to consider clinical importance, as indicated by using the Minimal Clinically Important Difference (MCID) [37]. Various studies indicated that a reduction of >2 mmHg in SBP and DBP could reduce coronary heart disease and stroke [38–39]. Researchers estimate a >2 mmHg reduction in SBP reduces CHD risk by 4%, stroke by 6%, and all-cause mortality by 3% [38]. When applying the >2 mmHg MCID criteria to this study’s findings, the present study reported an average decrease of 10 mmHg for SBP and an average decrease of 3 mmHg for DBP in the intervention group. Therefore, the reduction in blood pressure reported can be considered clinically important, resulting in a risk reduction.

When considering our results and approach to the design of the intervention program, the current study to some extent met the recommendations stipulated in a guideline document on the prevention of type 2 diabetes: Even a modest change in lifestyle that includes adopting a healthy diet, increasing PA and maintaining healthy body weight, may effectively prevent the risk for diabetes later in life, and these results have since been the basis for global prevention programs [40]. Hence, in this study, we did not follow a structured, gymnasium based aerobic and resistance program. While it is desirable, and perhaps essential to use some forms of structured exercise training as a medium to increase physical activity levels, these exercises might not be the exercises of choice for obese adolescents to continue over long periods. Therefore, the participants were involved in the design of the progressive aerobic component of the program to incorporate a variety of physical activities that can appeal to these obese adolescents. Due to the predominant Black ethnicity of the participants, traditional physical activities typical to their cultures like tag, modified shumpu, and ball games were included in the aerobic component of the program to allow them to develop their preferences and choices for the types of physical activities over the long term, which can help reduce sedentary behaviours in their lifestyles in adulthood. Despite keeping the program varied, exciting and fun, but still training within the set RPE levels to provide adequate benefits, the drop-out rate was 32% after 10-weeks to achieve a 65% adherence for the supervised and 67% for the unsupervised physical activity program and a 60% adherence for the nutritional education program. Systematic reviews and meta-analyses questions the optimal length of behaviour change interventions in obese adolescents [40]. Only studies six months and longer were included, with none of the reviews indicating the participants’ adherence to the intervention programs. In this regard, the authors initially set a 75% adherence target but, due to the reasons for dropping out (***Figure 1***), adherence was lowered to 65% as the CG continued with their activities of daily living (ADL) and were not sedentary and might have participated in physical activities.

The study’s findings have to be interpreted against some limitations experienced: First, the duration of the intervention program should have been longer, but due to the non-adherence of the participants, the intervention was stopped after 10 weeks instead of 16 weeks. Second, quantification of muscle mass and fat mass would have provided additional information to understand the significant decrease in BMI. Third, the control group’s ADLs translated to leisure-time physical activity were not measured with pedometers or accelerometers. Although the participants were asked to log their ADLs, a significant portion of the control neglected to record ADLs. Fourth, blood pressure measurement was not based on a series of measurements but on a single point in time.

## CONCLUSION

This study’s findings indicate that a 10-week physical activity and nutrition intervention improves body mass index and reduced blood pressure and selected biochemical variables in overweight and obese adolescents. In conclusion, we report that a relatively short physical activity and nutrition intervention may already show health benefits in overweight and obese children by improving body mass index and a clinically important reduction in blood pressure. Studies with a larger sample size addressing the reasons participants dropped out of the study should be conducted in the future.

## ADDITIONAL FILE

The additional file for this article can be found as follows:

10.5334/aogh.3147.s1 Effect of Physical Activity and Nutrition Education on Body Mass Index, Blood Pressure and Biochemical Variables in Overweight and Obese Adolescents.
